# Resveratrol promotes cholesterol efflux from dendritic cells and controls costimulation and T-cell activation in high-fat and lipopolysaccharide-driven atherosclerotic mice

**DOI:** 10.3389/fcvm.2024.1450898

**Published:** 2024-12-20

**Authors:** Linhui Zhang, Haixia Wang, Zishan Wang, Jianyi Xu, Mengyuan Wang, Wenxin Wang, Qiongshan He, Yun Yu, Dongping Yuan, Guirong Bu, Runze Qiu, Jun Long

**Affiliations:** ^1^ School of Pharmacy, Jiangsu Key Laboratory for Pharmacology and Safety Evaluation of Chinese Materia Medica, Nanjing University of Chinese Medicine, Nanjing, Jiangsu, China; ^2^ Department of Pharmacy, Wuxi Huishan Traditional Chinese Medicine Hospital, Wuxi, Jiangsu, China; ^3^ Department of Pharmacy, Nanjing First Hospital, Nanjing Medical University, Nanjing, Jiangsu, China

**Keywords:** atherosclerosis, resveratrol, dendritic cells, T cells, cholesterol efflux, ABCA1

## Abstract

Cholesterol aggregation in dendritic cells (DCs) triggers an inflammatory response and accelerates the development of atherosclerosis (AS). Resveratrol (RES), a natural compound with anti-inflammatory and cholesterol metabolism regulatory properties, has been shown to influence the maturation and inflammatory functions of DCs. However, its relationship with cholesterol metabolism remains unclear. This study aimed to explore the roles of RES in cholesterol metabolism and inflammatory behaviors of DCs in the context of AS. We analyzed the effect of RES on cholesterol efflux from ApoE^−/−^ bone marrow-derived dendritic cells (BMDCs) using qRT-PCR, Western blot, and cholesterol efflux assays; identified the inflammatory status of RES-treated BMDCs and co-cultured T cells using flow cytometry and ELISA; confirmed the effect of RES on blood lipids, atherosclerotic lesions, cholesterol metabolism, and inflammatory response in high-fat diet and lipopolysaccharide-treated ApoE^−/−^ mice; and explored the potential targets of RES in regulating inflammatory behavior via molecular docking. The results revealed that RES promotes cholesterol efflux, increases the expression of efflux transporter ABCA1, and decreases liver X receptor alpha (LXRα) expression in response to a decrease in intracellular cholesterol in ApoE^−/−^ BMDCs. RES also reduced MHC-II^+^ cells and downregulated costimulatory molecule CD80 in BMDCs with decreased IL-6 and increased IL-2 production, and suppressed T-cell activation and modulates IL-22 and IL-10 secretion via BMDCs. Furthermore, we confirmed that RES relieves arterial lesions and regulates blood lipids in ApoE^−/−^ mice. RES demonstrated ABCA1 upregulation and LXRα downregulation effects in the aorta and regulated costimulation molecules and Th17/Treg cytokines in the spleen. Furthermore, RES showed multiple hydrogen bonding and low binding energy with ABCA1, suggesting that ABCA1 is a potential target of RES to modulate the inflammatory properties of BMDCs. Our study demonstrated that RES regulates cholesterol efflux and inflammatory behavior in BMDCs, contributing to the control of AS progression and offering new insights for managing inflammatory diseases.

## 1 Introduction

Atherosclerosis (AS) is the basis for the pathogenesis of several cardiovascular diseases (1, 2) and is characterized by the formation of atherosclerotic plaque-containing immune cells (3). Antigen-presenting dendritic cells (DCs) play pivotal roles in the occurrence and development of AS (4, 5). Researchers have found that cholesterol metabolism can change the phenotype and function of DCs (6). In the lipid-enriched lesion intima, DCs absorb excess lipoproteins to form foam cells (7) and express elevated MHC-II, which bind to antigen peptides produced by proteolytic hydrolysis of self and non-self-proteins in endosomes and lysosomes, and present them to antigen-specific CD4^+^ T cells, thereby stimulating CD4^+^ T-cell activation and differentiation into T helper (Th) cell subsets. This process is critical to the progression of AS (8–11). Liver X receptor alpha (LXRα) signaling is a key mechanism for DCs to release cholesterol accumulated intracellularly (9). After being activated by endogenous oxidation and hydroxylation of cholesterol, LXRα promotes cholesterol efflux by upregulating ATP-binding cassette transporter (ABC)-A1 and G1 (7). However, under high-fat conditions, LXR expression in DCs is reduced, with elevated secretion of pro-inflammatory cytokines produced by activated NF-κB signaling (12, 13). Therefore, intervening in cholesterol efflux from DCs is important for controlling inflammatory responses and relieving plaque progression.

Resveratrol (RES) is a polyphenolic compound that is commonly found in plants such as *Vitaceae*, *Liliaceae*, *Polygonaceae*, *Leguminosae*, *Polygonum cuspidatum*, and *mulberry*. RES has been found to be associated with a reduced risk of AS (14, 15). RES has been observed to influence the atherosclerotic process by reducing endothelial cell apoptosis, dampening endothelial activation and vascular inflammation, and inhibiting the migration and proliferation of vascular smooth muscle cells, thereby exerting a broad effect on AS (16, 17). The regulation of cholesterol metabolism by RES has also been reported. RES can increase the expression of ABCA1/G1 and mediate cholesterol efflux macrophages, reducing the foaming macrophages (18, 19). DCs have different roles to macrophages in AS plaques, especially in T-cell response and differentiation. However, the role of RES in cholesterol efflux from DCs and its significance in alleviating atherosclerotic progression remain unclear.

In this study, we investigated the role of RES in cholesterol efflux, the inflammatory behavior of ApoE^−/−^ bone marrow-derived dendritic cells (BMDCs), and AS progression. The results suggest that RES can promote cholesterol efflux from DCs; control costimulatory effects, T-cell activation, and inflammatory responses; and alleviate AS, revealing its potential value in AS treatment.

## 2 Materials and methods

### 2.1 DC culture and administration

Six-week-old male ApoE^−/−^ mice were purchased from Beijing Vital River Laboratory Animal Technology Co., Ltd. (certificate number 11400700229437), housed in a specific pathogen-free (SPF) environment at the Nanjing University of Chinese Medicine [permit number SYXK (SU) 2018-0049], and euthanized by draining their blood after being anesthetized with enflurane. Bone marrow cells were extracted from the femur and tibia with phosphate-buffered saline (PBS) containing 0.1% bovine serum albumin (BSA) and 0.6% trisodium citrate, filtered through a 70-μm cell strainer, and purified using erythrocyte lysis buffer. The obtained cells were suspended in RPMI-1640 medium containing 10% fetal bovine serum (FBS), 100 units/ml penicillin, 100 μg/ml streptomycin, 1 mM sodium pyruvate, 50 μM 2-mercaptoethanol, 2 mM L-glutamine, and 10 ng/ml BMDC inducer GM-CSF and IL-4 (20) at 37°C and 5% CO_2_. All the medium was replaced on day 3, half of the medium was replaced on days 5 and 7, and 10 ng/ml GM-CSF and IL-4 were additionally added. BMDCs were collected on day 8 of incubation. All the cells were incubated with 5 μg/ml cholesterol (Solarbio, China) for 24 h except for the control (Ctrl) group. Furthermore, 1 mM cholesterol effector receptor sodium taurocholate (NaTC, TCI, Japan) was added to promote intracellular cholesterol outflow in the Ctrl and model groups (21). RES (Yuanye Biotechnology, China) was dissolved in 10% ethanol and an equal volume of 10% ethanol was used as the vehicle control.

### 2.2 T-cell preparation and co-culturing

C57BL/6J mice aged 6–8 weeks were purchased from Spearfish (Beijing) Laboratory Animal Technology Co., Ltd. (certificate number 1103241800005247), housed in an SPF environment at Nanjing University of Chinese Medicine [permit number SYXK (SU) 2018–0049], and were euthanized under enflurane anesthesia. The spleens were ground and filtered with a 100-μm cell filter, and the filtered cells were purified using erythrocyte lysis buffer. Spleen T cells were obtained using the Dynabeads Untouched Mouse T cells Kit (Invitrogen, USA). Briefly, after treatment with heat-inactivated serum, the cell suspension was labeled with an antibody mixture and beads to deplete B cells, monocytes/macrophages, natural killer (NK) cells, DCs, erythrocytes, and granulocytes using a magnetic rack. The remaining untouched T cells were resuspended in an RPMI-1640 medium containing FBS, penicillin/streptomycin, and L-glutamine. ApoE^−/−^ mouse BMDCs pretreated with cholesterol, NaTC, and RES were co-cultured with obtained total T cells at a ratio of 1:5 at 37 °C and 5% CO_2_ for 36 h, and 200 μg/ml ovalbumin was added as an antigen to stimulate antigen presentation. The upper layer of T cells was collected through a 70-μm cell filter and suspended in a serum-free medium for subsequent T-cell enzyme-linked immunosorbent assay (ELISA).

### 2.3 Animals

ApoE^−/−^ mice (6–7 weeks 18–22 g, male, certificate number 1400700352760) were provided by Beijing Vitong Lihua Laboratory Animal Technology Co., Ltd. and housed in an SPF barrier environment at the Laboratory Animal Center of Nanjing University of Chinese Medicine [permit number SYXK (SU) 2018–0049]. All animal experiments were conducted in strict accordance with the Guide for *the Care and Use of Laboratory Animals and Principles for the Use of Animals* issued by the National Institutes of Health (NIH) and approved by the Laboratory Animal Center of Nanjing University of Chinese Medicine.

After a 1-week acclimatization period, the mice were randomly assigned into four groups: Ctrl, high-fat diet (HFD) + lipopolysaccharide (LPS), simvastatin, and RES. The Ctrl group was fed a standard diet, while the other groups were fed an HFD containing 1.25% cholesterol and 20% lard for 20 weeks. In addition, all the mice except for those in the Ctrl group were given 10 μg/mouse of LPS (i.p., Sigma-Aldrich, USA) every 2 weeks starting in the 10th week. The mice in the simvastatin group were treated with 3.3 mg/kg simvastatin, and those in the RES group were treated with 5 mg/kg RES, dispersed in 5% sodium carboxymethyl cellulose solution daily for 20 weeks. At the end of the 20th week, the mice were weighed, fasted overnight, anesthetized with pentobarbital, and euthanized.

### 2.4 Cell counting kit-8 assay

After BMDCs were treated with 0, 5, 10, 20, 40, or 80 µM RES for 12, 24, or 48 h in 96-well plates (1 × 10^4^ cells/ml), the cytotoxicity was assessed by a cell counting kit-8 (CCK-8) reagent (APExBIO, USA, 10 µl per well). After incubation at 37°C for 2 h, the optical density (OD) value was recorded at 450 nm with a microplate reader (TECAN, Switzerland). The cell relative viability (%) was calculated as follows: (OD_RES, test_ − OD_RES, vehicle_)/(OD_0μM, test_ − OD_0μM, vehicle_) × 100%.

### 2.5 Cholesterol efflux assay

BMDCs were loaded with 10 μg/ml NBD-cholesterol (22-(N-(7-Nitrobenz-2-oxa-1, 3-Diazol-4-yl) amino) -23,24-bisnor-5-cholen-3β-ol) (Invitrogen, USA), a commonly used fluorescently conjugated cholesterol analog that can be internalized by cells such as cholesterol for 24 h in the dark (22). The medium was then removed and the cells were washed and treated with 5 and 10 µM RES, while the other cells were co-incubated in PBS containing 0.2% BSA for 24 h. At 18 h, 1 mM NaTC was added to the medium to induce cholesterol efflux. The medium and cells were collected using centrifugation and a 0.1 M NaOH solution was added to lyse in a vortex in the dark for 30 min. The lysate of the culture medium and cells was collected using centrifugation and added to a black 96-well plate. The fluorescence intensity (FI) of NBD-cholesterol in the medium were measured by a microplate reader with fluorescence (TECAN, Switzerland, excitation at 469 nm, emission at 537 nm). The cholesterol efflux ratio was calculated as follows: [FI_medium_/(FI_medium_ + FI_cell_)]_sample_/[FI_medium_/(FI_medium_ + FI_cell_)]_control_.

### 2.6 Flow cytometry

BMDCs were labeled with antibodies including MHC-II-BB700 (Invitrogen, USA), CD11c-FITC (BD Pharmingen, USA), CD80-APC (BD Pharmingen, USA), and CD86-PE (BD Pharmingen, USA), and resuspended in PBS with 1% BSA. After co-culturing with BMDCs, T cells were stained with antibodies including CD44-PerCP (BioLegend, USA) and CD62L PE MEL-14 (BD Pharmingen, USA) to assess their activation. The markers of BMDCs and T cells were then identified using a Beckman Coulter Gallios Flow Cytometer (Beckman Coulter, USA). Data were analyzed with FlowJo software (BD Biosciences, USA).

### 2.7 Enzyme-linked immunosorbent assay

The levels of IL-6 (EK2061/2), IL-2 (EK202HS-96), and TGF-β1 (EK2812/2) in the ApoE^−/−^ BMDCs medium and IL-17A (EK217HS-96), IL-22 (EK222HS-96), TGF-β1 (EK2812/2), and IL-10 (EK210/3-96) in the T cells co-cultured with BMDCs medium were determined using ELISA kits (Multisciences BioTECH, China).

### 2.8 Quantitative real-time PCR

BMDCs were treated with 3 μM LXR agonist T0901317 (MedChemExpress, USA) to amplify the expression of ABCA1 and ABCG1 for detection. TRIzol Reagent (Invitrogen, USA) was used to extract total RNA from BMDCs, aorta and spleen RNA (100 ng in 10 μl) was quantified with NanoDrop One (Thermo Scientific, USA), and 5X All-In-One RT MasterMix (ABM, Canada) was used to generate cDNA from mRNA in a Veriti 96-Well Thermal Cycler (Applied Biosystems, USA). Eva Green 2X qPCR Master Mix-Low ROX (ABM, Canada) was used for real-time qPCR with the ABI 7500 Real-time PCR system (Thermo Fisher Scientific, USA). Data were analyzed via the 2^−ΔΔCt^ method normalized to GAPDH. The primer sequences used are listed in Table 1.

**Table 1:** The list of specific primers used for qRT-PCR.

Gene	Primer (5′ to 3′)	
GAPDH	AGGTCGGTGTGAACGGATTTG(F)	TGTAGACCATGTAGTTGAGGTCA(R)
LXRα	CTCAATGCCTGATGTTTCTCCT(F)	TCCAACCCTATCCCTAAAGCAA(R)
ABCA1	AAAACCGCAGACATCCTTCAG(F)	CATACCGAAACTCGTTCACCC(R)
ABCG1	TCTGCGAATCACCTCGCACATT(F)	AGGGCAGCAAACATGAGGAACA(R)
CD80	ACCCCCAACATAACTGAGTCT(F)	TTCCAACCAAGAGAAGCGAGG(R)
CD86	AGTGATCGCCAACTTCAGTGAACC(F)	GGTGACCTTGCTTAGACGTGCAG(R)
IL-17A	CTCCACCGCAATGAAGAC(F)	CTCGACCCTGAAAGTGAAG(R)
IL-10	GCTCTTACTGACTGGCATGAG(F)	CGCAGCTCTAGGAGCATGTG(R)
IL-6	TAGTCCTTCCTCTACCCCAATTTCC(F)	TTGGTCCTTAGCCACTCCTTC(R)
IL-2	GCATGTTCTGGATTTGACTC(F)	ATTGAGGGCTTGTTGAGATG(R)

F, forward primer; R, reverse primer.

### 2.9 Western blotting

The total proteins in the BMDCs, aortas, and spleens were extracted via High-Efficiency RIPA Lysis Buffer (KeyGEN BioTECH, China) and quantified using a BCA protein concentration assay kit (Beyotime Biotechnology, China). The protein was separated by sodium dodecyl sulfate (SDS)-polyacrylamide gel electrophoresis and transferred onto a polyvinylidene fluoride membrane using an EPS-300 digital display stable voltage and current electrophoresis apparatus (TANON, China). The membranes were blocked with 5% BSA in Tris-HCl Tween buffer solution and incubated overnight at 4°C with antibodies against LXRα (Proteintech, China), ABCA1 (Affinity, USA), ABCG1 (Abcam, UK), and internal reference β-actin (Proteintech, China) or GAPDH (Proteintech, China). The membrane was then washed with TBST (TBS containing 0.1% Tween 20) buffer and incubated for 1 h with HRP-Goat Anti-Rabbit secondary antibody (H + L) (Proteintech, China) in blocking buffer. Finally, the Gel Doc XR + Gel Documentation System (Bio-Rad, USA) was used for immunodetection. The Image Lab 4.0 software was used to detect the gray values of the blots. The relative expression of the proteins was calculated by the formula Gray value_target protein_/Gray value_β-actin or GAPDH_.

### 2.10 Serum lipid analysis

The peripheral blood of the ApoE^−/−^ mice was collected from the posterior orbital venous plexus. The blood samples were centrifuged after standing, and the serum was collected for the analysis of blood lipids by total cholesterol (TC), triacylglycerol (TG), low-density lipoprotein cholesterol (LDL-C), and high-density lipoprotein cholesterol (HDL-C) detection kits (Jiancheng, China). The OD value of each well was measured with a microplate reader (TECAN, Switzerland). The concentrations of blood lipids were calculated using the standard curve method.

### 2.11 Arterial staining and pathological evaluation

The aortas of the ApoE^−/−^ mice were dissected from the aortic arch to the left and right common iliac artery branches, cut longitudinally along the ventral side, and nailed to a black foam board. After fixation with 4% paraformaldehyde (PFA), the plaques on the aortic intima were stained with Oil Red O powder (Solarbio, China) dissolved in isopropyl alcohol and the normal tissue was differentiated into milky white by 75% ethanol. The aortas were photographed and the stained plaque areas were measured with MapInfo 7.0 (USA, www.mapinfo.com). The ratio of aortic plaque to the total vessel area was calculated for each.

Mouse heart samples were fixed with 4% PFA, sequentially dehydrated with 50%, 70%, 85%, 95%, and absolute ethanol, and then paraffin-embedded. The paraffin blocks were sliced longitudinally perpendicular to the coronary artery, stained with hematoxylin and eosin, and photographed with a microscope (magnification ×400).

### 2.12 Molecular docking

The structure of RES was input as a ligand, and the protein structure bound to it was considered a potential target. The protein structure of the potential target of RES was downloaded from the The Research Collaboratory for Structural Bioinformatics (RCSB) Protein Data Bank (PDB) database (https://www.rcsb.org/), and dehydrated and deliganded via software PyMOL 2.4.0. The structure of RES was downloaded from the Traditional Chinese Medicine Systems Pharmacology (TCMSP) database (https://old.tcmsp-e.com/tcmsp.php/) and converted using OpenBabel 3.1.1 software. The structures of RES and its potential target were processed by AutoDock 4.2.6 software to run molecular docking and calculate the bond length and binding energy. The results were visualized using PyMOL 2.4.0 software.

### 2.13 Statistics

Data are presented as the mean ± standard deviation (SD) and were analyzed using GraphPad Prism 8 software. One-way analysis of variance was applied for comparisons between multiple groups and Tukey’s test was used for the *post-hoc* analysis. *P* < 0.05 was considered statistically significant.

## 3 Results

### 3.1 RES promotes NaTC-mediated cholesterol efflux from BMDCs

**Figure 1:**
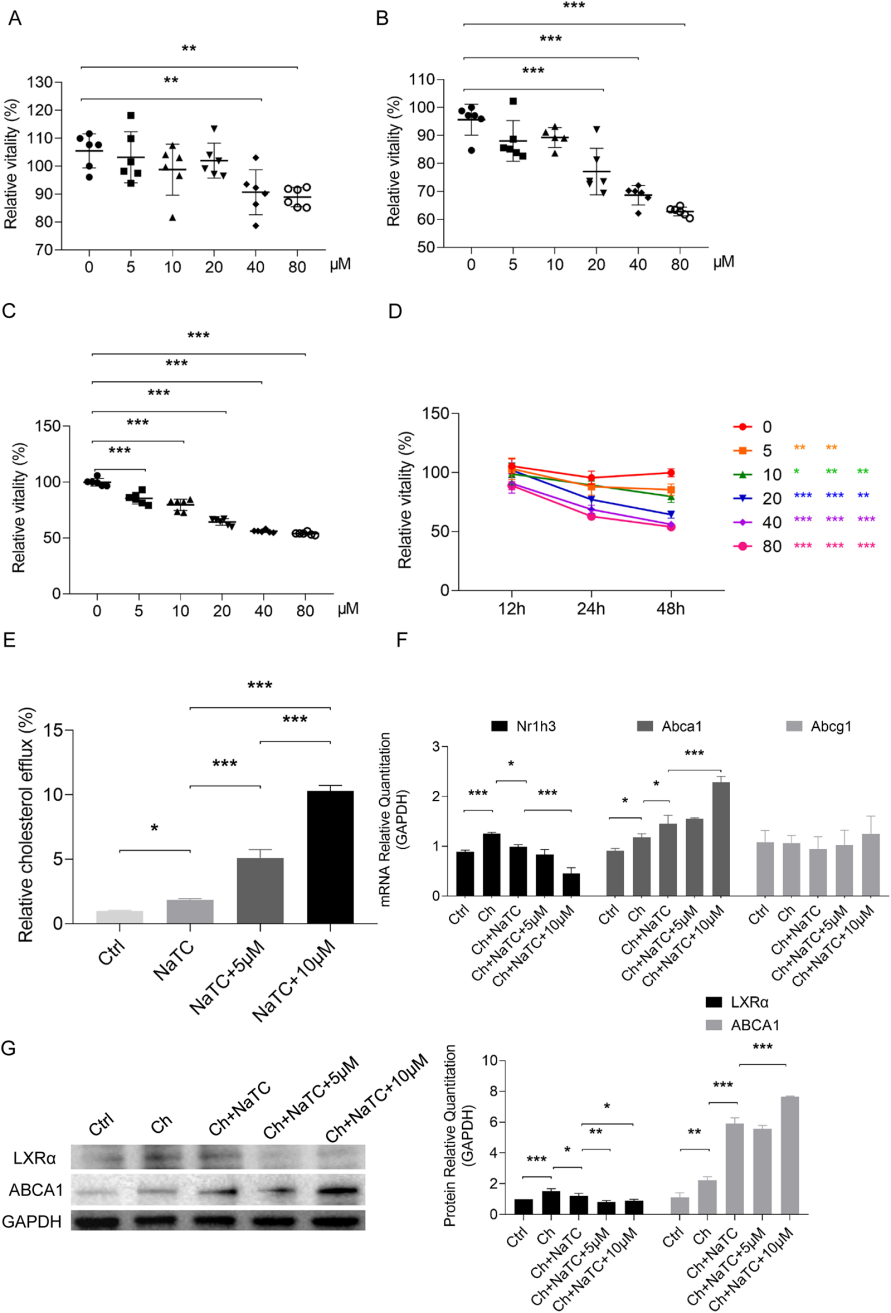
Effects of resveratrol on the growth and cholesterol efflux of BMDCs. **(A–D)** The proliferative viability of ApoE^−/−^ BMDCs after 12, 24, and 48 h treatment with different concentrations of resveratrol detected by CCK-8 assay. **(E)** NBD-cholesterol (ch) efflux of BMDCs treated with efflux recipient NaTC with or without different concentrations of resveratrol. **(F)** mRNA expression of genes related to ch efflux in BMDCs treated with resveratrol as measured by qRT-PCR. **(G)** Protein expression of genes related to ch efflux in BMDCs treated with resveratrol as measured by Western blotting. Data are represented as mean ± SD. *P < 0.05; **P < 0.01; ***P < 0.001.

To determine the safe dose of RES for ApoE^−/−^ BMDCs, dose-response tests were performed (Figures 1A–D). When the BMDCs were treated with RES for 12 h, a reduction in cell viability was observed at concentrations of 40–80 μM (Figure 1A), and RES at concentrations of 20–80 μM inhibited cell viability when BMDCs were treated for 24 h (Figure 1B). RES at concentrations of 5–80 μM showed cytotoxicity for the BMDCs treated for 48 h (Figure 1C). In addition to dose, the cytotoxicity of RES to BMDC also demonstrated a time-dependent nature (Figure 1D). To minimize toxicity, we selected 5–10 μM of RES for 24 h for subsequent experiments. Next, we conducted a cholesterol efflux experiment in NaTC-mediated ApoE^−/−^ BMDCs using NBD-cholesterol. Under the action of NaTC, NBD-cholesterol was released from BMDCs, and RES dose-dependently promoted NaTC-mediated cholesterol efflux from BMDCs (Figure 1E).

To explore the role of RES in cholesterol efflux from ApoE^−/−^ BMDCs at the molecular level, we examined the expression of key genes involved in cholesterol efflux, including Abca1 and Abcg1, which mediate cholesterol outflow and their upstream signal Nr1h3 (transcribing LXRα) (23, 24). In the presence of cholesterol, Nr1h3 and Abca1 expression increased, but no significant change was observed in Abcg1. After NaTC was added, Abca1 was further upregulated, while Nr1h3 expression decreased with the outflow of cholesterol. RES promoted an increase in Abca1 expression and a decrease in Nr1h3, especially at a dose of 10 μM (Figure 1F). We further detected changes in the protein levels of LXRα and ABCA1 and the results were consistent with mRNA changes, except that both 5 and 10 μM decreased LXRα expression based on NaTC-mediated cholesterol efflux (Figure 1G).

### 3.2 RES inhibits MHC-II expression and costimulation of BMDC and regulates the secretion of cytokines involved in Th-17 and regulatory T-cell differentiation in the cholesterol efflux environment

**Figure 2:**
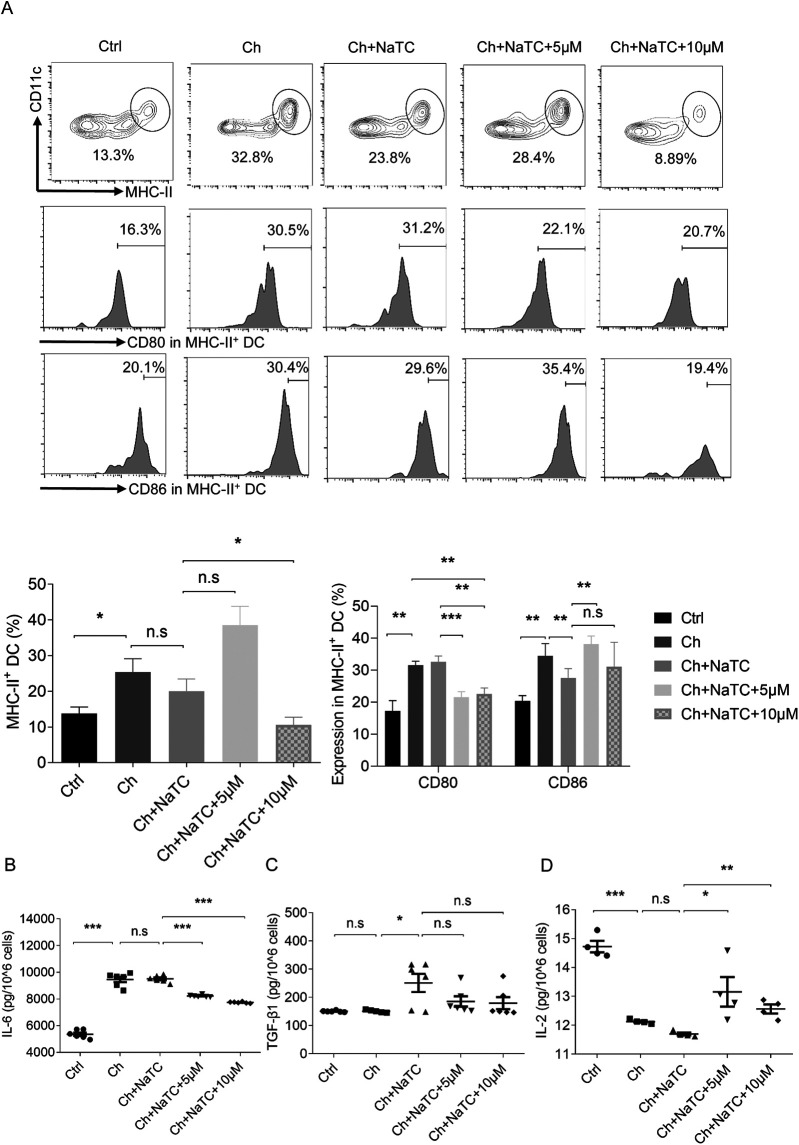
Effects of resveratrol on the number, costimulation, and cytokine secretion of dendritic cells. **(A)** The percentage of ApoE^−/−^ BMDCs co-labeled with MHC-II and CD11c and the percentage of BMDCs expressing costimulation molecules CD80 and CD86 detected by flow cytometry after being treated with cholesterol (ch), NaTC, and 0, 5, or 10 μM resveratrol. **(B–D)** IL-6, TGF-β1, and IL-2 levels in the supernatant of BMDCs treated with ch, NaTC, and 0, 5, or 10 μM resveratrol as assessed by ELISA. Data are represented with mean ± SD. *P < 0.05; **P < 0.01; ***P < 0.001.

The cholesterol metabolism of DCs affects their maturity, which subsequently affects the immune activation capacity of DCs (25). MHC-II in DCs mediates the antigen presentation to the T-cell receptors of CD4^+^ T cells, and costimulatory molecules such as CD80 and CD86 expressed at elevated levels in mature DCs is the second signal that mediates T-cell activation (26). To explore whether RES has an effect on these segments while inducing cholesterol efflux, we conducted the next experiment in the presence of cholesterol and NaTC. The number of MHC-II^+^ CD11c^+^ ApoE^−/−^ BMDCs was dramatically increased by cholesterol, accompanied by the upregulation of CD80 and CD86. The addition of NaTC reduced CD86 levels but had no effect on the number of MHC-II^+^ BMDCs and CD80 levels. The 5 μM RES dose weakly stimulated cholesterol efflux reduced CD80 expression but increased CD86 levels and promoted the proliferation of MHC-II^+^ BMDCs. When the dose was increased to 10 μM, the stimulating effect of CD86 was counteracted and the level of MHC-II^+^ BMDCs was reduced, while the inhibitory effect on CD80 remained (Figure 2A).

The balance between CD4^+^IL-17A^+^ Th17 and CD4^+^CD25^+^Foxp3^+^ regulatory T (Treg) cell responses is crucial in the pathogenesis of AS (27). Th17 cells, which are one of the main populations that mediate inflammatory responses, are counterbalanced by Treg cells, which secrete suppressive cytokines to maintain immune homeostasis and tolerance. Progressive accumulation of cholesterol in the ApoE^−/−^ BMDCs led to enhanced signals for the differentiation of Th17 responses (9, 28). This is due to the pivotal role of cytokines such as IL-6 and TGF-β1 in Th17 differentiation (29, 30). In contrast, TGF-β1, together with IL-2, facilitates the differentiation of Treg cells (31). The results showed that cholesterol-treated ApoE^−/−^ BMDCs secreted more IL-6 and less IL-2 (Figures 2B,D). No effect of NaTC on IL-6 and IL-2 levels was observed, but the release of TGF-β1 was promoted (Figure 2C). The 5–10 μM RES doses decreased secretion of IL-6 and increased IL-2 in cholesterol-loaded ApoE^−/−^ BMDCs in the presence of NaTC, but no further effect was observed on TGF-β1 levels. Overall, RES showed the potential to regulate the signals of T-cell activation and the balance between Th17/Treg differentiation in ApoE^−/−^ BMDCs.

### 3.3 BMDCs treated with RES attenuate T-cell activation and inflammatory cytokine secretion

**Figure 3:**
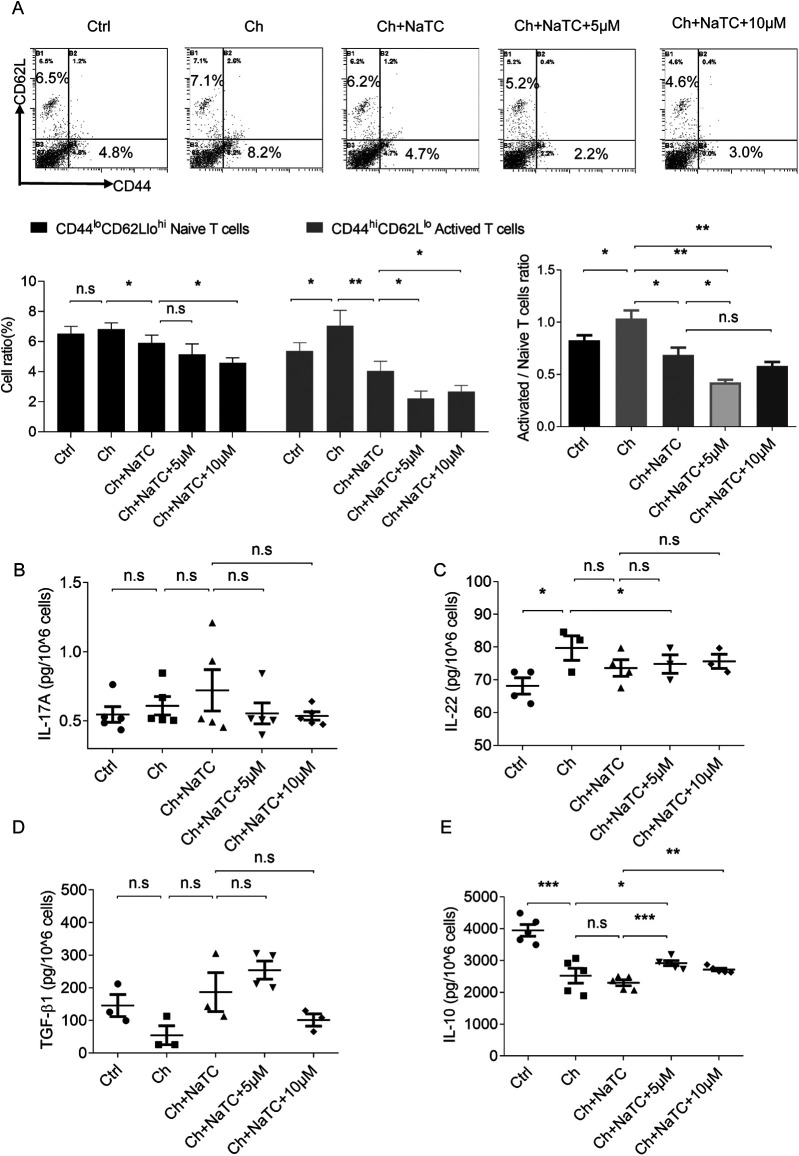
Effects of resveratrol-treated BMDCs on T-cell activation and cytokine secretion. **(A)** The percentage of CD44^lo^ CD62L^hi^ naive T cells and CD44^hi^ CD62L^lo^ activated T cells co-cultured with ApoE^−/−^ BMDCs treated with cholesterol (ch), NaTC, and 0, 5s or 10 μM resveratrol as detected by flow cytometry. **(B–E)** The levels of IL-17A, IL-22, TGF-β1, and IL-10 in the supernatant of T cells after co-culturing as determined by ELISA. Data are represented with mean ± SD. *P < 0.05; **P < 0.01; ***P < 0.001.

Mature DCs effectively activate naïve T cells by presenting antigens and providing costimulatory signals, playing a central role in initiating and maintaining immune responses (32). Given the changes observed in the signals responsible for T-cell activation and differentiation in ApoE^−/−^ BMDCs, we further investigated whether RES could affect T-cell activation via BMDCs in the presence of cholesterol. The aggregation of cholesterol in BMDCs increased the number of CD44^hi^CD62L^lo^ activated T cells during the presentation of antigen ovalbumin, which was reversed by NaTC-mediated cholesterol efflux. The 5–10 μM RES doses further amplified the inhibition of NaTC-treated BMDCs on activated T cells (Figure 3A).

Activated T cells can modulate the immune response by secreting different cytokines. Within the CD4^+^ lineage, Th17 cells produce pro-inflammatory cytokines such as IL-17 and IL-22, while Tregs secrete suppressive cytokines such as TGF-β and IL-10 (33). We found that cholesterol-loaded ApoE^−/−^ BMDCs caused an increase in the secretion of IL-22 and a decrease in IL-10 from antigen-activated T cells but did not alter IL-17A and TGF-β1 levels. NaTC-mediated BMDC cholesterol efflux did not further affect the release of these cytokines from T cells. RES-treated BMDCs upregulated the IL-10 level secreted by T cells in the presence or absence of NaTC, and decreased IL-22 and increased TGF-β1 levels in synergy with NaTC (Figures 3B–E). These results suggest that RES-treated BMDCs inhibit T-cell activation and inflammatory behavior by inducing cholesterol efflux.

### 3.4 RES alleviates dyslipidemia and arterial lesions in ApoE^−/−^ mice

**Figure 4:**
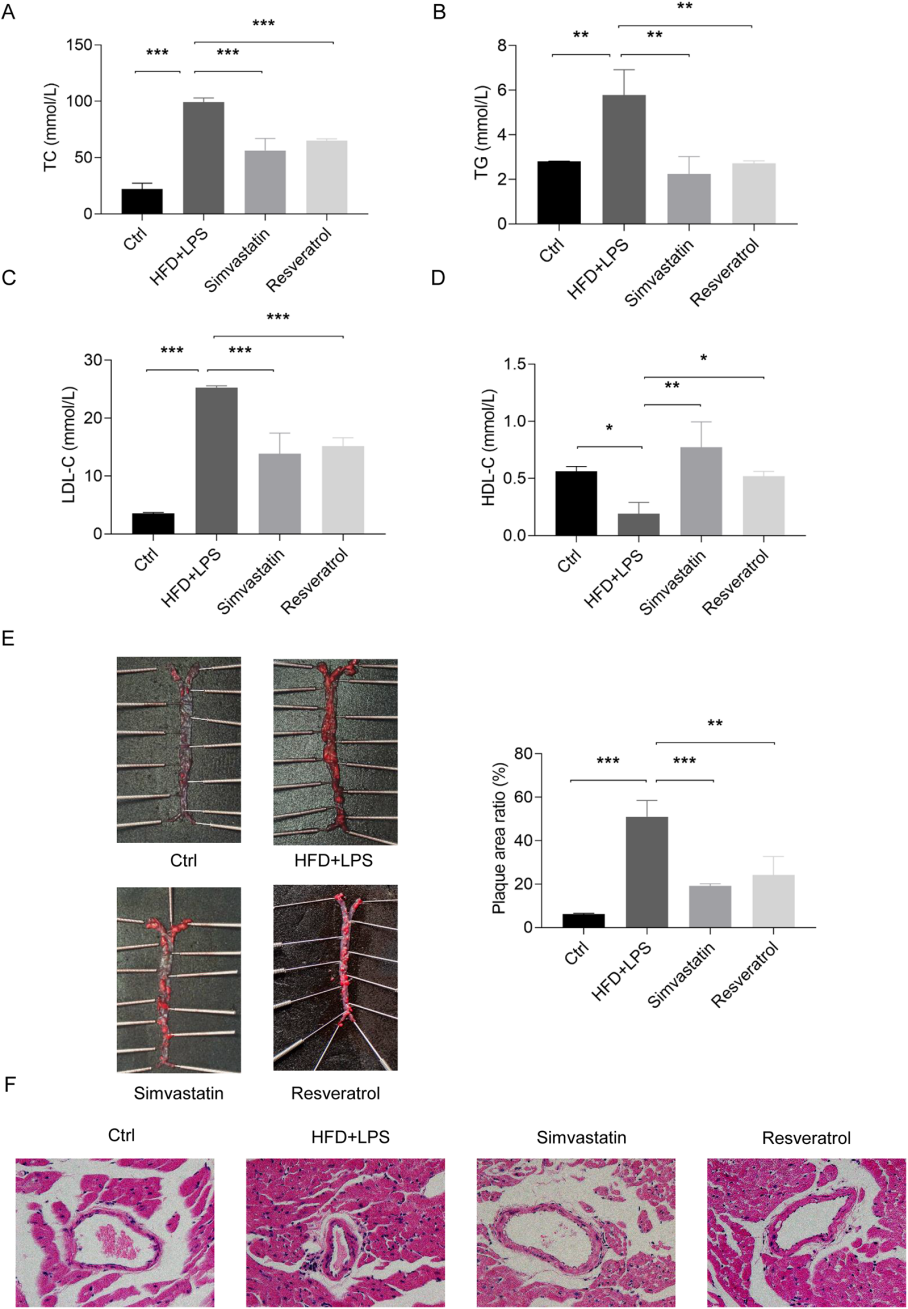
Effects of resveratrol on blood lipids and atherosclerotic lesions in the ApoE^−/−^ mice treated with a high-fat diet (HFD) and LPS. **(A–D)** The levels of TC, TG, LDL-C, and HDL-C in the serum of ApoE^−/−^ mice treated with an HFD and LPS and administered simvastatin or resveratrol. **(E)** Aortic plaque in mice as assessed by Oil Red O staining. **(F)** Coronary artery lesions in mice as assessed by H&E staining (magnification: × 400). Data are represented with mean ± SD, *P < 0.05, **P < 0.01, ***P < 0.001.

LPS is a component of the outer membrane of the cell wall of gram-negative bacteria and induces immune activation and accelerates the development of AS by mimicking infectious inflammatory patterns (34, 35). To assess the role of RES in AS, we constructed a model using an HFD and LPS administration in ApoE^−/−^ mice, an animal model characterized by lipid metabolism disorders. In this model, mice treated with an HFD and LPS exhibited elevated levels of serum TC, TG, and LDL-C, along with reduced levels of HDL-C. In contrast, oral administration of the lipid-lowering drug simvastatin and RES both effectively corrected this dyslipidemic profile (Figures 4A–D). An HFD and LPS also caused severe aortic plaque and inflammatory infiltration and thickening of the coronary artery wall in the ApoE^−/−^ mice (Figures 4E,F). RES treatment significantly inhibited aortic plaque formation, reduced inflammatory infiltration, and attenuated coronary artery stenosis, displaying effects similar to simvastatin. These findings demonstrate that RES has lipid-regulating and anti-atherosclerotic properties in HFD and LPS-induced atherosclerosis in mice.

### 3.5 RES activates cholesterol efflux, inhibits costimulation, and controls inflammatory cytokine secretion in mice with AS

**Figure 5:**
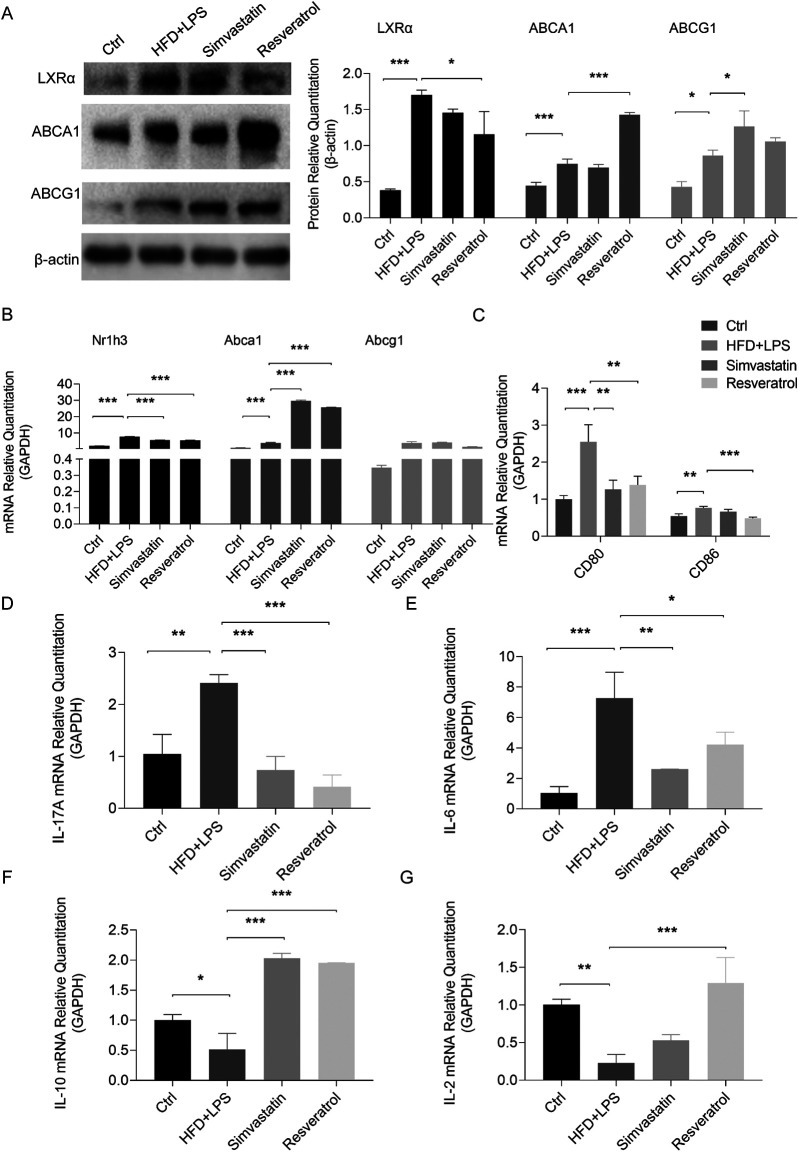
Effects of resveratrol on cholesterol efflux, costimulatory signaling, and cytokine expression in the HFD and LPS-treated ApoE^−/−^ mice. **(A,B)** Expression of proteins and mRNAs related to cholesterol efflux in the aortas of the HFD and LPS-treated ApoE^−/−^ mice administered with simvastatin or resveratrol as detected by Western blotting and qRT-PCR. **(C)** The mRNA levels of costimulatory molecules CD80 and CD86 in the spleen of mice as detected by qRT-PCR. **(D–G)** The mRNA levels of cytokines IL-17A, IL-6, IL-10, and IL-2 in the spleens of mice as detected by qPCR. Data are represented with mean ± SD. *P < 0.05; **P < 0.01; ***P < 0.001.

Atherosclerotic plaques contain a variety of cells that take up redundant lipids, such as smooth muscle cells (SMCs), T cells, DCs, and macrophages. Abnormal lipid metabolism in these cells is closely related to the development of AS (3). Given the role of cholesterol efflux in DC inflammatory behavior, we examined the expression of aortic genes and proteins related to cholesterol efflux. In the case of the mice fed an HFD and administered LPS, the expression of the LXRα (Nr1h3), ABCA1, and ABCG1 proteins increased, but the mRNA expression of Abcg1 did not change significantly. The administration of RES showed no significant effect on ABCG1, but further increased the expression of ABCA1, and the expression of LXRα was correspondingly decreased. In contrast, simvastatin, an antilipemic agent, primarily elevated ABCG1 expression rather than ABCA1 (Figures 5A,B).

Subsequently, we analyzed the expression of costimulatory molecules and cytokines in the spleen to understand the effect of RES on T-cell activation and differentiation in atherosclerotic mice. The results showed that RES reversed the increase in the transcription of CD80 and CD86 in the spleens of the ApoE^−/−^ mice stimulated by an HFD and LPS, while simvastatin only modulated the level of CD80 (Figure 5C). The HFD and LPS-mediated AS environment stimulated the transcription of the Th17 cytokines, IL-17A and IL-6, and downregulated the Treg cytokines, IL-10 and IL-2, in the spleens. These effects were reversed by RES, and simvastatin had no effect on IL-2 (Figures 5D–G).

### 3.6 ABCA1 is a potential target of RES to promote BMDC cholesterol efflux and ameliorate atherosclerosis

**Figure 6:**
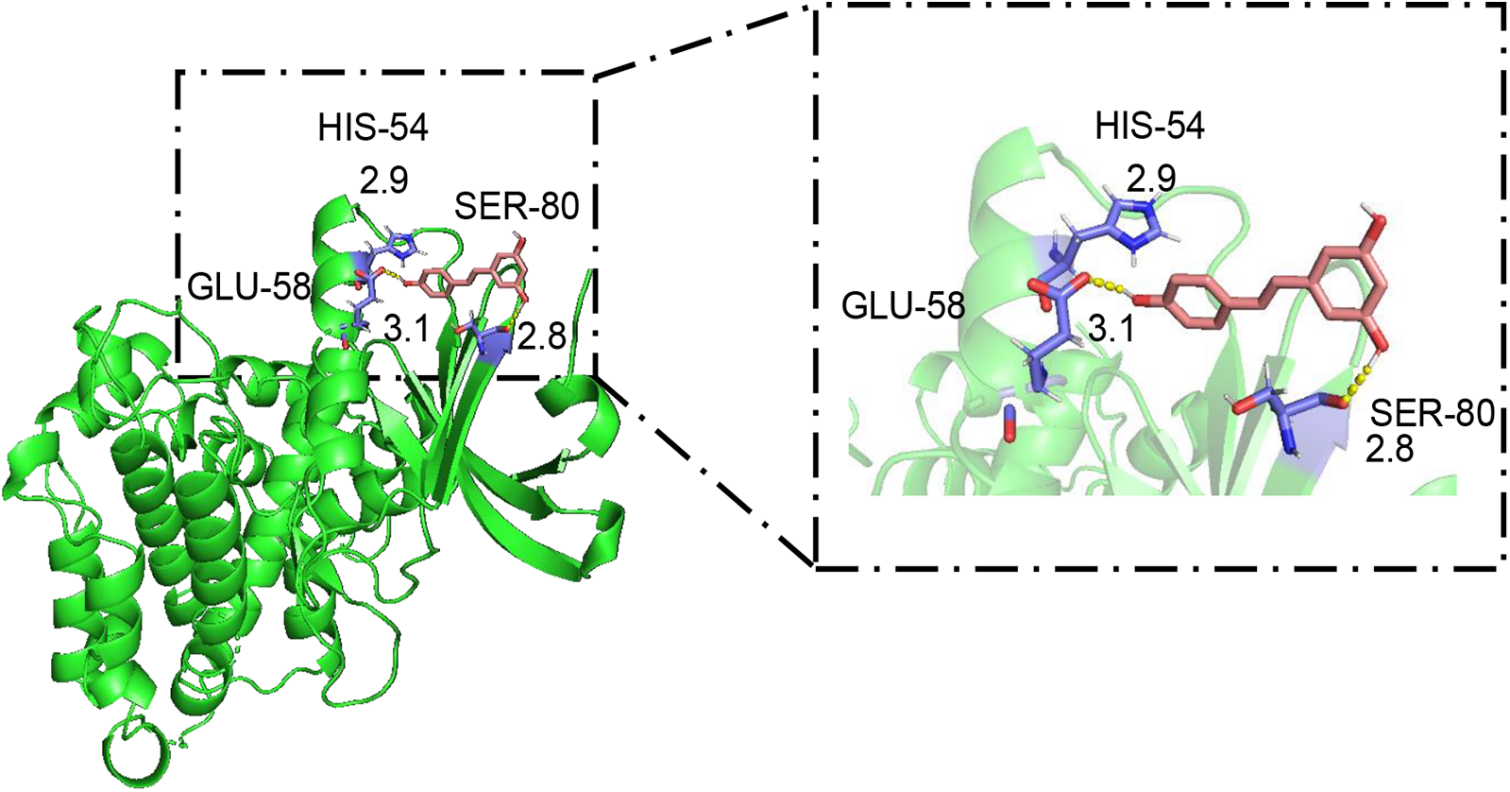
Molecular docking of resveratrol to ABCA1. In the binding model, the structures of resveratrol and ABCA1 are indicated by pink and green, respectively; the hydrogen bonds between resveratrol and ABCA1 are represented as yellow dashed lines; and the ligand binding sites are indicated in purple. The length (Å) of hydrogen bonding between RES and the amino acid residues of ABCA1 is indicated at the junction.

Considering the stimulating effect of RES on the cholesterol efflux transporter ABCA1 in BMDCs and the aortas of ApoE^−/−^ mice, we confirmed the binding capacity of RES to ABCA1 by molecular docking. RES forms hydrogen bonds with three amino acid residues of ABCA1, including GLU-58 (length 3.1 Å), HIS-54 (length 2.9 Å), and SER-80 (length 2.8 Å) (Figure 6), and the binding energy was quite low (−3.04 kcal mol^−1^). RES formed a strong interaction with ABCA1, suggesting that ABCA1 is a potential target of RES to regulate cholesterol efflux and inflammatory behavior of DCs in AS.

## 4 Discussion

AS is a lipid disorder characterized by arterial plaque and chronic inflammation. The accumulation of LDL in the intima matrix is the initializing event (36). Once oxidatively modified, LDL is phagocytosed by macrophages derived from monocytes to form foam cells, the main component of plaque (37, 38). In addition to elevated LDL levels, a decrease in HDL levels, which are responsible for the reverse transport of cholesterol, and an increase in TG are also characteristic of blood lipid profiles in AS patients, which synergistically promote the occurrence of cardiovascular incidents (39). RES is a natural compound derived from plants that has beneficial biological activities including anti-oxidation, anti-inflammation, and anti-apoptosis properties, exhibiting positive effects in several metabolic disorders (40). In terms of AS, RES has been previously reported to reduce lipid accumulation in macrophages by inducing cholesterol efflux to delay plaque formation (41). In our study, RES reduced TC, TG, and LDL-C levels, and restored HDL-C levels in the serum of the ApoE^−/−^ mice treated with HFD and LPS, and effectively reduced plaque formation and coronary artery lesions with effects comparable to the antilipemic agent simvastatin. It is worth noting that the highly efficient effect of RES on DCs may be associated with an improvement in AS.

DCs play a critical role in mediating innate and adaptive immune responses, which accumulate in arterial plaque and accelerate the development of AS (42). MHC-II, which is responsible for antigen presentation, is often used as an identifier of DCs in conjunction with CD11c (43, 44). CD80 and CD86 are the predominant phenotypes of mature DCs that mediate costimulation (45). In addition to macrophages, excess cholesterol in the arterial intima can be absorbed by DCs to enhance antigen presentation and inflammatory activity and accelerate AS progression (7). Furthermore, cholesterol efflux signaling regulates antigen presentation and pro-inflammatory behavior in DCs (25). Therefore, intervening in cholesterol metabolism in DCs is of great importance to delay or halt the development of AS. To investigate the effects of RES on DCs, we utilized BMDCs in our *in vitro* study, which were derived from bone marrow precursor cells and induced by GM-CSF and IL-4, with a high expression of MHC-II and CD80, a low expression of macrophage marker F4/80, and potent antigen-presenting and T-cell-activating capabilities (46, 47). Accordingly, we constructed a NaTC-induced cholesterol efflux model to explore the biological characteristics of BMDCs in the presence of cholesterol and the effects of RES. NaTC is a weak detergent capable of acting as an extracellular cholesterol receptor, whose activity to induce cholesterol efflux is comparable to ApoA-I (48, 49). Our results showed that RES promoted NaTC-mediated cholesterol efflux in ApoE^−/−^ BMDCs, which may be involved in the attenuation of the inflammatory response in AS.

Detecting changes in key signal levels is helpful for further understanding the regulatory patterns of RES on DC cholesterol efflux. LXRα mediates cholesterol efflux as an upstream signal of ABCA1 and ABCG1, playing an important role in cholesterol homeostasis (24). ABCA1 mediates the efflux of cholesterol and phosphatidylcholine to ApoA-I and NaTC, a lipid-free apolipoprotein generating nascent HDL (preβ-HDL) (50), whereas ABCG1 mediates the efflux of cholesterol, phosphatidylcholine, and sphingomyelin to nascent HDL and HDL (51, 52). ABCA1/G1 deficiency in DCs increases cholesterol accumulation and the inflammatory phenotype (9). We found that the expression of LXRα and ABCA1 were upregulated in a cholesterol-rich environment, and NaTC further upregulated the expression of ABCA1 but decreased the expression of LXRα in ApoE^−/−^ BMDCs. Studies have shown that LXRα is upregulated when intracellular cholesterol aggregates (53). Our results reveal that the expression of LXRα and its downstream signal ABCA1 were elevated after the addition of cholesterol, and LXRα was decreased by NaTC with the efflux of intracellular cholesterol. RES, with cholesterol efflux-stimulating activity, further increased the expression of ABCA1 while decreasing LXRα expression in BMDCs and the aortas of ApoE^−/−^ mice. The BMDCs and ApoE^−/−^ mice treated with RES showed no significant effect on ABCG1, which may be due to their greater tendency to efflux cholesterol toward a lipid-free carrier. In contrast, the antilipemic agent simvastatin upregulated the expression of ABCG1 but not ABCA1 in the mouse aortas, suggesting that it is more inclined to outflow cholesterol to HDL.

To further explore how RES combines with ABCA1, we tested their intermolecular interactions. From the molecular docking results, RES shows multiple hydrogen bonding and low binding energy with ABCA1, suggesting that ABCA1 is a potential target of RES to control DCs cholesterol efflux to NaTC and arterial plaque progression.

As mentioned above, the interaction of DCs with CD4^+^ T cells determines the development of AS. As tissue sentinels, DCs continually sample antigens from their local environment and process them for presentation to CD4^+^ or CD8^+^ T cells on MHC-II or MHC-I molecules, respectively (54). The complete T-cell response also requires the participation of the “second signal,” the costimulation generated by the interaction of molecules such as CD80 and CD86 on the surface of mature DCs with CD28 and other molecules on the surface of T cells. DCs can also create a microenvironment that enables naïve T cells to differentiate into effector T cells by releasing cytokines, which is also known as the “third signal” (55, 56). IL-6 and TGF-β1 are responsible for the differentiation of inflammatory Th17 cells characterized by the expression of transcription factor nuclear receptor ROR*γ*t (RORC) and the secretion of the inflammatory factors IL-17 and IL-22 (57). TGF-β1 can also induce the differentiation of immunosuppressive Tregs together with IL-2 (58). IL-2 stimulation can cause Tregs to produce high levels of IL-10, which inhibits the formation of atherosclerotic lesions (57). Our results showed that the high-cholesterol environment stimulated the proliferation, costimulatory molecule expression, and T-cell activation of MHC-II^+^ BMDCs. The cytokine profiles in the microenvironment tilted to the Th17 cells, including upregulated IL-6 and downregulated IL-2 in DCs, and elevated IL-22 and reduced IL-10 secreted by co-cultured T cells. A similar phenomenon also appeared in the ApoE^−/−^ mice treated with an HFD and LPS. RES inhibited the T-cell activation signals in BMDCs when promoting NaTC-induced cholesterol efflux, and transferred cytokine secretion to induce Tregs, downregulating IL-6 while upregulating IL-2, resulting in decreased IL-22 secretion and increased IL-10 release from T cells, which may be involved in delaying AS progression.

CD80 and CD86 provide distinct costimulatory effects on T cells. CD86 might play a key role in the early decision-making stage of immune activation, while CD80 is related to the expansion of late-activated T cells and the persistence of the responses (59). The inhibition of costimulation by RES in BMDCs was mainly concentrated in CD80 and even had a weak stimulating effect on CD86 transcription at 5 μM, which disappeared with the increase in cholesterol efflux stimulation. During this *in vivo* study, we found that both simvastatin and RES inhibited the transcription of CD80 in the spleen, but RES showed additional inhibition on CD86, which may be related to other antigen-presenting cells, such as macrophages.

## 5 Conclusion

In general, RES induces the expression of the cholesterol transporter ABCA1 and enhances cholesterol efflux in DCs while inhibiting the proliferation, costimulation, and T-cell activation of DCs, and tilts the cytokine microenvironment toward a Treg-dominated anti-inflammatory pattern. In the ApoE^−/−^ mice treated with an HFD and LPS, RES ameliorated dyslipidemia and atherosclerotic lesions in the aortas and coronary arteries, upregulated the expression of ABCA1 in the aortas, and reduced the levels of costimulatory molecules and inflammatory cytokines in spleens and serum. RES showed a strong interaction with ABCA1, suggesting a potential target for regulating cholesterol efflux and inflammatory responses. The characteristics of RES that stimulate cholesterol efflux from DCs to alleviate their inflammatory behavior make this natural compound a potential agent for the treatment of AS.

## Data Availability

The original contributions presented in the study are included in the article/Supplementary Material, further inquiries can be directed to the corresponding authors.
